# Network analysis of psychological resilience, personality traits, and depression–anxiety–stress symptoms among military college students in China

**DOI:** 10.3389/fpsyt.2026.1684090

**Published:** 2026-01-29

**Authors:** Yanqin Hou, Guimin Zhang, Mingdi Mi, Buyao Wang, Tingwei Feng

**Affiliations:** 1 Department of Health Management and Services, Cangzhou Medical College, Hebei, China; 2 School of Education, Hebei University, Hebei, China; 3 Student Affairs Office, Weinan Vocational and Technical College, Weinan, China; 4 Mental Health Education and Consultation Center, Tarim University, Alaer, China; 5 Department of Military Medical Psychology, Air Force Military Medical University, Xian, Shanxi, China

**Keywords:** big five, bridge centrality, DASS-21, military academy students, network analysis, personality traits, psychological resilience

## Abstract

**Background:**

Non-military-status undergraduate cadets in Chinese military academies face a distinctive combination of academic demands and militarized stressors, which may increase psychological distress and shape resilience-related adaptation. Although personality traits are associated with psychological resilience, systematic evidence is still lacking regarding how resilience and personality jointly relate to depression–anxiety–stress symptoms in quasi-military contexts.

**Methods:**

This cross-sectional study examined the associations among psychological resilience, Big Five personality traits, and depression–anxiety–stress symptoms in 855 non-military-status undergraduate cadets. Participants completed the 10-item Connor–Davidson Resilience Scale (CD-RISC-10), the Chinese Big Five Personality Inventory (CBF-PI-15), and the Depression Anxiety Stress Scales–21 (DASS-21). Network estimation was performed using graphical LASSO with Spearman partial correlations. Centrality indices, including bridge strength and bridge expected influence (BEI), were computed to identify key nodes and their roles in the overall network structure.

**Results:**

The gender-stratified CD-RISC-10–CBF-PI–DASS-21 networks were relatively dense, with 19/36 and 25/36 non-zero edges and similar mean edge weights. Network comparison tests indicated significant differences in overall structure (*M* = 0.12, *p* <.001) and global strength (3.89 vs. 4.04; *S* = 1.15, *p* = .01), suggesting tighter coupling among resilience, personality, and distress indicators in females. In both networks, resilience exhibited the most prominent bridging role, whereas agreeableness functioned as a shared protective bridge node. The strongest positive bridge edge differed by gender. Bridge metrics showed good stability, and bootstrap confidence intervals supported the accuracy of edge-weight estimates.

**Conclusion:**

This study provides clinically informative, network-based evidence on psychological adaptation in a rarely examined cadet population. The central bridging role of resilience, the protective bridging role of agreeableness, and gender-specific trait–resilience linkages offer actionable, empirically grounded targets for stratified psychological interventions and resilience-promotion programs in high-demand, quasi-military training settings. These findings have important implications for psychological support strategies and mental health promotion in military education environments.

## Highlights

- This study integrates resilience, Big Five traits, and DASS-21 distress symptoms within a single regularized partial-correlation network in non-military-status cadets.
- Gender-stratified network comparison showed significant differences in network structure and global strength, with tighter overall connectivity in females.
- Resilience consistently emerged as the core bridge node linking personality traits and distress indicators, whereas Agreeableness functioned as a shared protective bridge across genders.
- The strongest positive bridge pathway differed by gender: Conscientiousness–Resilience was most salient in males, whereas Neuroticism–Resilience was most salient in females.
- Network estimates demonstrated acceptable accuracy and stability (CS = 0.64–0.75), supporting the robustness of the observed bridge patterns.

## 1 Introduction

Under the unique institutional framework of military academies in China, non-military-status undergraduate cadets experience a distinctive set of stressors. In addition to the academic demands faced by all college students, this group is also subjected to the rigorous demands of militarized management and discipline, which markedly differ from the environment of civilian universities (1, 2). These compounded pressures and the specific context in which they occur play a pivotal role in shaping the psychological states and adaptive mechanisms of cadets during their undergraduate years (3, 4).

Despite the growing research on psychological resilience and personality traits among general college populations, studies focusing specifically on military academy undergraduates—particularly those without formal military status—remain scarce (5, 6). This group occupies a unique transitional identity between civilian and military life, offering a valuable lens through which to explore individual differences in psychological adaptation under structured, high-pressure conditions (7). As such, investigating their resilience and personality configurations holds considerable academic and applied significance (8).

This study focused on non-military-status undergraduate cadets in Chinese military academies, investigating the current status of their psychological resilience and Big Five personality traits (9). Utilizing network analysis, we constructed a network structure model to examine the interrelations between psychological resilience and personality traits (10). From a symptom-level network perspective, the study aimed to characterize the network structure and conditional association patterns between these constructs, with the goal of providing a theoretical foundation and intervention strategies to enhance psychological resilience in high-pressure environments (11, 12).

Research indicates that the Big Five personality traits—extraversion, openness, conscientiousness, neuroticism, and agreeableness—may influence an individual’s psychological resilience (13). Campbell-Sills and Cohan et al. found that resilience is negatively correlated with neuroticism and positively correlated with extraversion (14). Following this, Makaya et al. supported these findings and further revealed positive correlations between resilience and both openness and conscientiousness (15). Annalakshmi confirmed that all factors of resilience are associated with adjustments in personality traits (16). Early studies also demonstrated that resilient children show enhanced reasoning and problem-solving abilities, along with greater intellectual behavior and comprehension skills (17). Annalakshmi analyzed the relationship between psychological resilience and personality traits, suggesting that traits such as self-exposure, impulsivity, and comprehension influence the development of psychological resilience, while resilience factors can, in turn, affect personality traits (18). Additionally, research conducted by the University of the West Indies found that healthy personality traits play a crucial role in the psychological resilience of Caribbean adolescents; such traits contribute to improved well-being, whereas unhealthy traits are linked to decreased well-being (19). This finding is echoed in the research by Deng Huihui et al. on college students in Guizhou, which similarly indicates that personality traits can predict individual psychological resilience (20). Resilience has been conceptualized as a trait-based psychological capacity (21), a dynamic process of positive adaptation (22), and a multisystem construct encompassing biological, psychological, and social systems (23).

Personality, encompassing both innate dispositions and characteristics shaped through life experiences, can be defined as “a distinctive pattern of factors that influence an individual’s emotions, thoughts, and behaviors, thereby differentiating one person from another” (24). These enduring traits contribute to substantial individual differences in psychological responses to traumatic events, such as those triggered by the COVID-19 pandemic (25). Among military college students, the interplay between personality and psychological adjustment is particularly salient (26). This population is required not only to navigate typical developmental tasks associated with emerging adulthood but also to adapt to the stringent academic, behavioral, and physical demands imposed by a militarized institutional environment (27). Such dual-role expectations amplify the psychological burden on cadets, making the cultivation and maintenance of psychological resilience an essential component of their successful adaptation and overall well-being (28).

The relationship between personality factors and psychopathological symptoms is complex (29), and this relationship may be bidirectional: psychopathological symptoms can vary due to differences in personality traits, while personality traits may also be influenced by the presence of psychopathological symptoms. The Big Five personality model posits that individual differences in normal behavior can be categorized into five independent dimensions: neuroticism, extraversion, openness, agreeableness, and conscientiousness (30). Although the Big Five model is widely recognized as an important framework for describing personality (31, 32), it may not adequately capture the entire range of normal personality variation, and the number of traits it encompasses could be insufficient for making significant predictions about various phenomena, including psychopathological symptoms (33).

In addition, depression–anxiety–stress symptoms constitute an important symptom-level marker of psychological adaptation and risk. The Depression Anxiety Stress Scales–21 (DASS-21) is a widely used instrument with a clear three-factor structure and demonstrated psychometric soundness across diverse populations, including Chinese college samples. Accumulating evidence indicates robust links between the Big Five and negative emotional symptoms: Neuroticism is consistently associated with higher levels of depression, anxiety, and stress, whereas traits such as Extraversion, Agreeableness, and Conscientiousness tend to be associated with lower distress (26). Psychological resilience is likewise inversely related to psychological distress and may buffer the impact of stress exposure on symptom expression, potentially functioning as a protective resource within broader personality–distress pathways (34). However, under quasi-military conditions characterized by rigid discipline and heightened performance demands, the relative “weights” of these links may be reconfigured, and the bridge pathways connecting personality traits, resilience, and distress symptoms remain insufficiently characterized using network approaches.

In recent years, advancements in network science have prompted researchers to employ network structural analysis methods to delve into the relationship between psychological resilience and personality traits among university students. This approach not only uncovers the intricate and nuanced connections between psychological resilience and personality traits but also identifies core factors and key nodes within this extensive relational network. By conducting meticulous analyses, researchers aspire to accurately identify specific personality traits that significantly impact the psychological resilience of university students. This insight will provide a scientific foundation and effective entry points for developing targeted interventions aimed at enhancing the psychological resilience of this demographic.

In network analysis, traits are represented as nodes, while the relationships between these traits are illustrated through edges connecting the nodes, with thicker edges indicating stronger associations. Network graphs offer a visual representation of the interconnected traits and highlight core traits within the network. In studies exploring personality and psychopathology, three centrality measures are commonly employed to assess the significance of nodes (35): strength, closeness, and betweenness. Strength refers to the total sum of correlations between a node and other nodes in the network and is typically considered the most relevant metric. Closeness is defined as the reciprocal of the distance between a node and other nodes in the network. Betweenness quantifies how frequently a node serves as the shortest path between two other nodes, thus reflecting its importance in maintaining connections within the network. When dark personality traits exhibit high levels of strength, closeness, and betweenness, they are likely to be considered core traits. Furthermore, in cases where negative associations exist between nodes, a fourth centrality measure—expected influence —is calculated, which accounts for these negative relationships (36). The use of network analysis in psychopathological research is becoming increasingly prevalent for illustrating the relationships among groups of psychiatric symptoms (37). However, its application to personality traits remains relatively limited, although such applications have demonstrated the potential to enhance our understanding of personality (38).

Personality traits have a significant impact on individuals’ perceptions and evaluations of their work environments, and conversely, the work environment can also influence an individual’s personality traits (39). Personality is not only a crucial factor in the development of stress but also alters the degree to which stress affects an individual (40). Depending on their personality characteristics, people may demonstrate varying levels of sensitivity and resilience to stress (41–43). In our research, we identified a significant positive correlation between the neuroticism dimension, nurse stress, and fear of COVID-19. Existing literature indicates that individuals with neurotic personality types are more likely to experience negative emotional states and stress (44, 45). As a result, nurses with neurotic personality traits may be more adversely affected both physically and psychologically, encountering higher levels of stress.

Emerging research indicates that psychological resilience—a collection of traits reflecting an individual’s toughness—is a critical factor in shaping health and well-being during later life (46, 47). This evidence suggests that individuals who cultivate and maintain certain traits and strategies throughout their lives may draw on these characteristics and strategies to promote their health as they age, particularly when facing challenges. However, the predictive validity of psychological resilience as a personal resource has not been fully established when compared to other known resources that influence health and well-being, such as mastery, optimism, and despair (48). Within personality traits, conscientiousness and neuroticism have been identified as factors influencing fear of COVID-19, while conscientiousness, neuroticism, and openness to experience have an impact on psychological resilience (49). The tested model demonstrated a good fit and elucidated the direct effects of the variables under study.

Nonetheless, this study encounters several challenges and issues. First, the relationship between psychological resilience and personality traits may be influenced by a variety of factors, including cultural background, social environment, and personal experiences. These influences complicate the relational network and increase the complexity of the research. Second, as a relatively new research method, network structural analysis is still in the exploratory phase of its application in psychology, requiring further refinement and validation in terms of its theoretical framework, analytical techniques, and interpretive capacity. Moreover, converting research findings into practical and effective intervention strategies to meaningfully enhance the psychological resilience of university students remains a critical focus area for ongoing research.

This research presents two major academic innovations: First, it focuses on a rarely studied yet highly applicable population within psychological research—non-military-status cadets in military academies. Second, by employing network analysis, the study establishes a structural bridge between personality traits and symptom-level processes, thereby advancing the theoretical framework of psychological resilience from a trait-oriented approach to a more intervention-focused perspective.

To address this research gap, we have constructed a network structure that integrates psychological resilience and personality traits and have examined the characteristics of this network. The primary objectives of this study are threefold: (1) to investigate the relationships between psychological resilience and the various items and dimensions of personality; (2) to identify the most critical central nodes within the entire network; and (3) to determine key bridging nodes that facilitate the positive or negative influence of the Big Five personality traits on psychological resilience. With these aims, we aspire to generate more in-depth, detailed, and comprehensive research outcomes that provide solid support for enhancing psychological resilience and personality health among university students.

## 2 Materials and methods

### 2.1 Participants

The data for this study were collected via paper-and-pencil surveys administered on campus between January 16 and April 18, 2023. A total of 897 undergraduate cadets participated; 42 questionnaires were excluded due to incomplete demographic information or failure to pass two embedded honesty-check items. Consequently, the final analytic sample comprised 855 valid questionnaires (valid response rate = 95.3%), including 412 males and 443 females, with a mean age of 19.10 years (SD = 1.42). All participants were officially enrolled undergraduate cadets in Chinese military academies with non-military status. Their identity and enrollment status were confirmed prior to data collection through institutional records and class rosters provided by academic offices, following established procedures for participant verification in military academy research (50, 51). Only cadets without formal military status were eligible to participate. In addition, demographic information and two honesty-check items embedded in the questionnaire further ensured data validity and sample eligibility. These procedures guaranteed that the final sample comprised exclusively verified non-military-status cadets. The current study was reviewed and approved by the Medical Ethics Committee of the First Affiliated Hospital of the Fourth Military Medical University (No. KY20222135-C-1). The study was conducted in accordance with the Declaration of Helsinki guidelines. After reading the informed consent, participants can complete the following survey if they want to further participate in this study. We will try to protect participants’ privacy.

### 2.2 Measurements

#### 2.2.1 10-item Connor–Davidson resilience scale

The CD-RISC-10 is a widely used self-report questionnaire designed to evaluate resilience across various populations, including adolescents, elderly individuals, and psychiatric patients. Each item is rated on a 5-point scale from 0 “not true at all”to 4 “true nearly all the time”, with higher scores indicating a greater ability to handle adversity. The Chinese version was utilized in this study, demonstrating excellent internal consistency (α = 0.94) (52). Although the CD-RISC-10 primarily reflects the perceived capacity for resilience, it has been recognized as a reliable proxy of adaptive functioning in both student and military contexts.

#### 2.2.2 The Chinese big five personality inventory

The scale was created by Dr. Wang Mengcheng and Professor Dai Xiaoyang as a simplified version of CBF-PI. It includes a total of 15 items across five dimensions and utilizes a six-point Likert scale, where 1 indicates “strongly disagree” and 6 indicates “strongly agree.” The scale exhibits satisfactory reliability and validity, with psychometric properties that outperform similar instruments both domestically and internationally. The internal consistency coefficient is 0.79. In the present sample, internal consistency for the CBF-PI-15 subscales (three items per trait) was acceptable to excellent: Extraversion (α = 0.81), Neuroticism (α = 0.93), Openness (α = 0.81), Agreeableness (α = 0.90), and Conscientiousness (α = 0.90) (53).

#### 2.2.3 The depression anxiety and stress scale

The scale comprises a total of 21 items, with each subscale of depression, anxiety, and stress consisting of 7 items. Participants rate each item on a 4-point Likert scale ranging from 0 (does not apply) to 3 (applies completely), with higher total scores indicating more severe negative emotions. In China, numerous studies have been conducted to evaluate the psychometric properties of the DASS-21. Gong were the first to introduce the Chinese version of this scale and conducted a survey among college students, demonstrating its stable psychometric properties and its ability to reflect the psychological distress experienced by Chinese college students. The DASS-21 has been widely used in various countries and populations, including China, and has shown good reliability and validity. The DASS-21, developed by Lovibond, has demonstrated good internal consistency, with Cronbach’s alphas of 0.94 for depression, 0.87 for anxiety, and 0.91 for stress (54). With a Cronbach’s alpha coefficient of 0.90.

### 2.3 Statistical analysis

The data analysis comprised two components: descriptive statistics and network analysis. Initially, we conducted descriptive statistical analysis on all data using SPSS version 23.0. This involved computing means, standard deviations, and Cronbach’s α coefficient. Subsequently, network modeling of the data was performed using RStudio software (version 4.3.1) (55), where we calculated Bridge Strength and Bridge Expected Influence (BEI). The procedures for network analysis followed standardized guidelines as outlined by Epskamp et al. (2018) (56), encompassing five main aspects: network estimation, visualization of the network, centrality indices, network accuracy assessment and stability estimation.

This study explored two networks: psychological resilience and personality. The network structures of both undirected networks were estimated using graphical lasso network methods. In each network, edges represent the partial correlation between two nodes while controlling for the influences of all other remaining nodes. The networks were constructed based on Spearman partial correlations. To regularize the partial correlations represented within the networks, the graphical least absolute shrinkage and selection operator (LASSO) technique was applied. This technique penalizes very small partial correlation coefficients to zero, thereby aiding in the removal of spurious edges and resulting in more stable and sparse networks. The extended Bayesian Information Criterion (EBIC) hyperparameter γ was set to 0.5 to achieve a balance between sensitivity and specificity. The networks presented were based on the Fruchterman-Reingold algorithm, with the qgraph package in R utilized to compute these networks (57).

This study predefined two communities: the psychological resilience community and the Big Five personality community (which includes Extraversion, Neuroticism, Openness, Agreeableness, and Conscientiousness). To identify bridge nodes connecting these communities, the bridge expected influence was calculated, defined as the sum of the edge weights connecting a given node to all nodes in other communities. Measures of bridge centrality (such as bridge strength and betweenness) are particularly effective for identifying bridge nodes within positive connection networks. A higher bridge expected influence value suggests a greater likelihood of activating the opposing community. The expected influence of bridges was calculated using network analysis tools in the R package.

The accuracy of the edge weights was evaluated by plotting the 95% confidence intervals for each edge, utilizing 2000 bootstrap samples. To assess the stability of the bridge expected influence, we employed a case-drop bootstrap method (also with 2000 bootstrap samples) to calculate the correlation stability (CS) coefficient. Following established guidelines, an ideal CS coefficient is expected to be greater than 0.5 and should not fall below 0.25. Additionally, we conducted a bootstrap difference test on the edge weights and bridge expected influences, again using 2000 bootstrap samples. All procedures were carried out using the R package “bootnet (58).”

#### 2.3.1 Network estimation

Using the R software’s qgraph package (57), we conducted partial correlation network estimation on the overall item dimensions of the sample. Circular nodes represent different dimensions or items, and the edges connecting the nodes reflect the strength of the partial correlation coefficients, with thicker edges indicating stronger correlations. Each partial correlation network underwent Gaussian graphical model (GGM) estimation (59).

#### 2.3.2 Visualization of the network

Visualization estimations were conducted separately for networks of psychological resilience. The *Fruchterman-Reingold* algorithm was utilized for network layout (55). In this study, positively correlated edges were depicted in blue, while negatively correlated edges were shown in red. The thickness of the edges reflected the strength of associations between symptoms/variables. The *averageLayout* function from the R qgraph package was employed for network layout configuration (50).

#### 2.3.3 Centrality Indices

In this study, we employed the Bridge Expected Influence Index (BEI) (60), which is a centrality metric used to quantify the coreness of nodes within a network structure, revealing the importance of nodes in the overall network. A higher Expected Influence Index of a node indicates greater influence within the network.

#### 2.3.4 Network accuracy assessment and stability estimation

In this study, we assessed the accuracy and stability of network estimates using the bootnet package in R (61). The accuracy of edge weights was evaluated using 95% bootstrap confidence intervals around the bootstrapped edge weights. A narrower confidence interval indicates more accurate edge estimates. We utilized the Centrality Stability Coefficient (CS) to assess stability.

## 3 Results

### 3.1 Descriptive statistics

The average (SD) age of the undergraduate individuals, ranging from 16 to 26, was 19.10 (1.42) years old. (As shown in **Table 1**).

**Table 1:** The means, standard deviations and bridge expected influences of the items in the Resilience-Personality-DASS-21 network.

Items	Abbreviation	Male			Female		
		M	SD	BEI	M	SD	BEI
10-item Connor–Davidson Resilience Scale (CD-RISC-10)							
Resilience	R	28.69	8.41	0.57	29.22	6.44	0.47
The Chinese Big Five Personality Inventory (CBF-PI)							
Neuroticism	Neu	11.84	3.30	0.26	11.74	2.99	0.27
Conscientiousness	Con	12.63	3.00	0.32	12.61	2.66	0.24
Agreeableness	Agr	10.71	2.23	-0.11	10.67	2.17	-0.19
Openness	Ope	10.42	2.54	0.01	10.10	2.35	-0.01
Extraversion	Ext	10.68	2.52	0.11	10.70	2.31	0.16
The Depression Anxiety and Stress Scale (DASS-21)							
Anxiety	Anx	3.86	0.26	0.01	3.39	0.20	-0.01
Depression	Dep	3.27	0.50	0.01	3.04	0.21	0.01
Stress	Str	3.01	0.97	-0.01	2.35	0.42	0.01

M, mean; SD, standard deviation; BEI, bridge expected influence.

To examine sex differences, we conducted independent-samples t-tests comparing the male group and the female group across the nine dimensions (Neu, Con, Agr, Ope, Ext, Resilience, Anxiety, Depression, and Stress). The results showed that males scored significantly higher than females on Openness (10.42 ± 2.54 vs. 10.10 ± 2.35; *t* = 6.48, *p* <.001, effect size =0.13), Anxiety (3.86 ± 2.26 vs. 3.39 ± 2.00; *t* = 11.06, *p* <.001, effect size=0.22), Depression (3.27 ± 2.50 vs. 3.04 ± 2.21; *t* = 4.90, p<.001, effect size=0.10), and Stress (3.01 ± 2.97 vs. 2.35 ± 2.42; t=12.33, *p* <.001, effect size =0.25). In contrast, females reported significantly higher Resilience than males (29.22 ± 6.44 vs. 28.69 ± 8.41; *t* = -3.52, *p* <.001, effect size=-0.07). No significant sex differences were observed for Neuroticism, Conscientiousness, Agreeableness, or Extraversion.

### 3.2 Network analysis

#### 3.2.1 The CD-RISC-10-CBF-PI-DASS-21 network

In the cross-sectional comparison of the CD-RISC-10–CBF-PI–DASS-21 item network between the two groups, both networks exhibited relatively high density. The first-group network comprised 9 nodes with 19 non-zero edges (19/36), whereas the second-group network comprised 9 nodes with 25 non-zero edges (25/36). The mean edge weights were 0.074 and 0.072, respectively, indicating that non-zero associations were common among CD-RISC-10, CBF-PI, and DASS-21 indicators in both groups. Network invariance testing showed a significant difference in overall network structure between groups (M = 0.12, p < 0.001), suggesting that the specific pattern of connections among items was not identical across the two populations. Global strength invariance testing further indicated that global strength differed significantly between the two networks (3.89 vs. 4.04; S = 1.15, p = 0.01), with the second-group network demonstrating greater overall connectivity. This finding implies that, in the female group, the interrelations among resilience, Big Five personality traits, and depression–anxiety–stress indicators were more tightly coupled, reflecting a higher degree of interdependence across nodes. In contrast, the male network appeared relatively more loosely connected, with greater structural independence among dimensions.

In the CD-RISC-10–CBF-PI–DASS-21 network, the male group (Group 1) showed the strongest positive bridging association for the Conscientiousness–Resilience (Con–R) edge (Male = 0.32; Female = 0.24). In contrast, in the female group network, the Neuroticism–Resilience (Neu–R) edge exhibited the strongest positive bridging association (Female = 0.27; Male = 0.26). In addition, within the female network, the Resilience–Agreeableness (R–Agr) edge showed the strongest negative bridging association (Female = −0.19; Male = −0.17).

**Figure 1:**
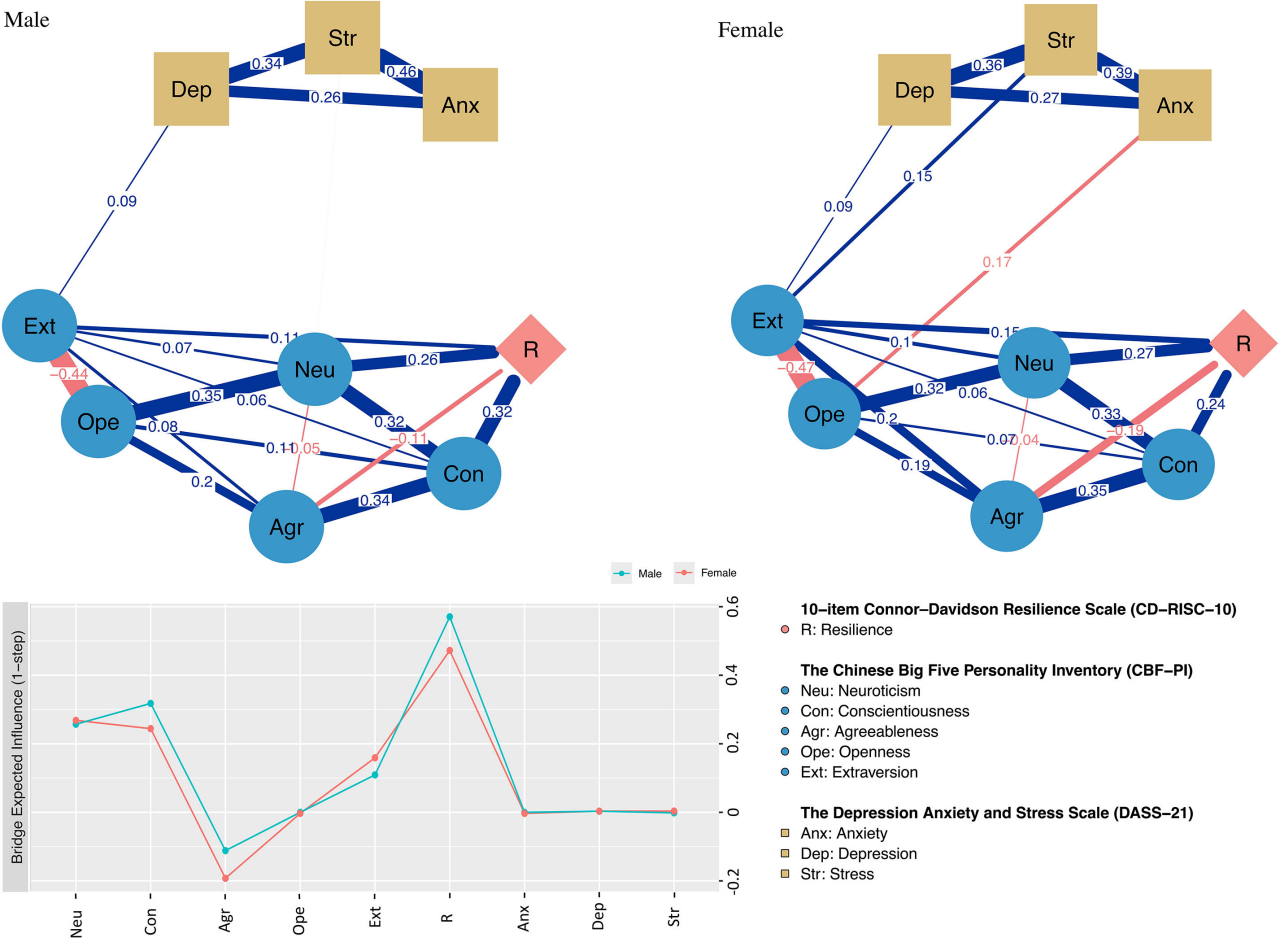
An estimated network model for CD-RISC-10-CBF-PI-DASS-21 item in the total sample. Blue edges represent positive correlations between the two nodes, while red edges represent negative correlations. The thickness of the edges reflects the magnitude of the correlation. The ring around the nodes depicted its predictability. Centrality plot, of the Resilience-Personality-depression–anxiety–stress items, shown as a standardized values z scores. BEI, bridge expected influence (1-step).

In the male network (**Figure 1**, left panel), the strongest positive associations were Con–R (weight = 0.32), Neu–R (weight = 0.26), Extraversion–Resilience (Ext–R) (weight = 0.11), and Extraversion–Depression (Ext–Dep) (weight = 0.09). The strongest negative association was Agreeableness–Resilience (Agr–R) (weight = −0.11).

In the female network (**Figure 1**, right panel), the strongest positive associations were Neu–R (weight = 0.27), Con–R (weight = 0.24), Ext–R (weight = 0.15), and Extraversion–Stress (Ext–Str) (weight = 0.15). The strongest negative associations were Agr–R (weight = −0.19) and Openness–Anxiety (Ope–Anx) (weight = −0.17).

The correlation stability (CS) coefficients were 0.64 for the male network and 0.75 for the female network, indicating good stability of the estimated bridge expected influence (BEI) in the CD-RISC-10–CBF-PI–DASS-21 networks. Overall, the network analyses revealed group differences in both network structure and bridging patterns across CD-RISC-10, CBF-PI, and DASS-21 indicators. Resilience (R) emerged as a shared risk-related bridge node in both groups (Male BEI = 0.57; Female BEI = 0.47), whereas Agreeableness (Agr) functioned as a shared protective bridge node (Female BEI = −0.19; Male BEI = −0.11). Taken together, both networks demonstrated acceptable structural stability, supporting the robustness of the findings.

## 4 Discussion

### 4.1 The resilience–personality–distress network

By embedding DASS-21 symptoms into the resilience–personality framework, this study provides a symptom-relevant map of psychological adaptation in non-military-status cadets under quasi-military demands. The networks suggest that resilience, personality traits, and depression–anxiety–stress indicators are conditionally interconnected rather than separable domains (62). Across sexes, resilience showed the most prominent bridging role linking trait dispositions to distress symptoms, whereas agreeableness consistently exhibited a protective bridging pattern (negative BEI), implying that affiliative tendencies may dampen cross-domain activation between traits and distress (63, 64). This integration strengthens interpretability beyond trait–resource associations by clarifying how these constructs relate to symptom-level distress (65).

### 4.2 Why traits act as risk or protective factors

From a theoretical perspective, two mechanisms may explain these context-specific effects. First, the demand–control–effort pathway clarifies why Conscientiousness may transform from an adaptive trait into a liability under rigid structures. Excessive self-discipline and perfectionistic striving, though facilitating performance, may increase strain and reduce psychological recovery, leading to its central risk role in the network (66). Second, the flexibility–cohesion pathway explains the protective influence of Openness and Agreeableness. Openness fosters cognitive flexibility and adaptive coping, while Agreeableness enhances prosociality and peer support. These characteristics buffer negative affect and promote resilience in group-based, high-stress environments (67). This dual mechanism interpretation situates our results within broader trait–context interaction theories and highlights the importance of considering sociocultural settings in resilience research (68).

### 4.3 Practical implications for cadet psychological support

Trait effects appear context-dependent. Although conscientiousness is typically protective in civilian samples, under rigid, high-demand training conditions it may carry costs through overcontrol, perfectionistic striving, and reduced recovery, which can make it a risk-relevant connector even when positively related to resilience (69, 70). In contrast, openness (cognitive flexibility) and agreeableness (prosocial support and cohesion) are plausibly protective in group-based, high-stress settings and align with the observed protective cross-domain patterns (66–68). These findings underscore that quasi-military structures may “reweight” trait functioning relative to general populations (71).

### 4.4 Methodological considerations and future directions

The sex-stratified network comparison adds an additional layer that cannot be inferred from mean differences alone. Although some mean-level contrasts were modest, the network comparison suggested that females showed greater overall connectivity (global strength) and a different structural configuration. One plausible interpretation is that, among female cadets, resilience, personality, and distress may be more tightly coupled—so shifts in one domain (e.g., distress) are more likely to co-occur with changes in others (e.g., resilience resources). In males, the comparatively lower connectivity may reflect greater compartmentalization among domains. These are not claims about causality, but they are meaningful for how psychological risk might “cluster” in practice.

The bridge-edge patterns further suggest sex-specific leverage points. The most salient positive bridge edge involved Conscientiousness–Resilience in males, whereas Neuroticism–Resilience was most salient in females (72). This does not imply that conscientiousness is “bad” for males or that neuroticism is the “cause” of lower resilience for females; rather, it highlights where the strongest conditional coupling sits in each network and thus where intervention may gain traction. For male cadets, it may be important to preserve the performance benefits of conscientiousness while reducing its potential costs (e.g., rigid standards, reduced recovery, excessive self-monitoring). For female cadets, interventions that combine resilience skills with techniques that directly target neuroticism-linked processes—rumination, threat sensitivity, negative appraisal bias—may be especially relevant. Across both groups, the consistent bridge role of resilience supports resilience-focused programming (distress tolerance, cognitive reappraisal, flexible persistence), while the shared protective bridging role of agreeableness supports peer-based approaches (mentoring, cohesion-building, structured help-seeking channels). bridges and connectivity patterns that can guide hypothesis-driven intervention development.

## 5 Limitations

Another limitation of this study is the absence of a civilian college student control group. Without such a comparison, it is not possible to conclude definitively whether the observed network patterns are unique to non-military-status cadets or reflect broader resilience–personality associations common to young adults. While our findings provide valuable insights into a rarely examined population, future studies should incorporate general college samples to validate the specificity and generalizability of the present results. Because the data are cross-sectional, the estimated network represents contemporaneous conditional associations and cannot establish causal directionality or temporal (cross-lagged) relations between resilience and personality; longitudinal and cross-lagged network models are needed to test directionality over time.

## 6 Conclusion

In a large sample of non-military-status undergraduate cadets in Chinese military academies, we used network analysis to characterize the conditional association structure among psychological resilience, Big Five personality traits, and depression–anxiety–stress symptoms (DASS-21). Across sexes, resilience emerged as the most influential bridge node linking personality traits to distress indicators, whereas agreeableness showed a consistently protective bridging pattern. Conscientiousness occupied a central position and may represent a context-sensitive risk-relevant connector under rigid, high-demand training conditions, while openness showed comparatively protective cross-domain connectivity. Network comparison further indicated significant sex differences in overall structure and global strength, suggesting tighter coupling among resilience, traits, and distress in female cadets. Collectively, these findings provide a network-informed framework that can guide targeted, resilience-focused psychological support and symptom prevention strategies in quasi-military educational settings.

## Data Availability

The original contributions presented in the study are included in the article/supplementary material. Further inquiries can be directed to the corresponding author.
